# Immune profiling reveals umbilical cord blood mononuclear cells from South India display an IL-8 dominant, CXCL-10 deficient polyfunctional monocyte response to pathogen-associated molecular patterns that is distinct from adult blood cells

**DOI:** 10.1093/cei/uxae034

**Published:** 2024-05-02

**Authors:** Vasista Adiga, Hima Bindhu, Asma Ahmed, Nirutha Chetan Kumar, Himanshu Tripathi, George D’Souza, Mary Dias, Sudarshan Shivalingaiah, Srishti Rao, Shanti K N, Catherine Hawrylowicz, Pratibha Dwarkanath, Annapurna Vyakarnam

**Affiliations:** Human Immunology Laboratory, Division of Infectious Diseases, St. John’s Research Institute, Bangalore, Karnataka, India; Department of Biotechnology, PES University, Bangalore, Karnataka, India; Human Immunology Laboratory, Division of Infectious Diseases, St. John’s Research Institute, Bangalore, Karnataka, India; Human Immunology Laboratory, Division of Infectious Diseases, St. John’s Research Institute, Bangalore, Karnataka, India; Human Immunology Laboratory, Division of Infectious Diseases, St. John’s Research Institute, Bangalore, Karnataka, India; Human Immunology Laboratory, Division of Infectious Diseases, St. John’s Research Institute, Bangalore, Karnataka, India; Department of Pulmonary Medicine, St. John’s Medical College, Bangalore, India; Division of Infectious Diseases, St. John’s Research Institute, Bangalore, Karnataka, India; Division of Infectious Diseases, St. John’s Research Institute, Bangalore, Karnataka, India; Division of Infectious Diseases, St. John’s Research Institute, Bangalore, Karnataka, India; Department of Biotechnology, PES University, Bangalore, Karnataka, India; Peter Gorer Department of Immunobiology, School of Immunology & Microbial Sciences, Faculty of Life Science & Medicine, King’s College, London, UK; Division of Nutrition, St. John’s Research Institute, Bangalore, Karnataka, India; Human Immunology Laboratory, Division of Infectious Diseases, St. John’s Research Institute, Bangalore, Karnataka, India; Peter Gorer Department of Immunobiology, School of Immunology & Microbial Sciences, Faculty of Life Science & Medicine, King’s College, London, UK

**Keywords:** toll-like receptors, pathogen-associated molecular patterns, monocytes, neonates, cytokines

## Abstract

Neonate responses to pathogen-associated molecular patterns (PAMPS) differ from adults; such understanding is poor in Indian neonates, despite recognized significant infectious risk. Immune profiling analysis was undertaken of 10 secreted mediators contextualized with cellular source induced by six PAMPs in umbilical cord (CB; *n* = 21) and adult-blood (PBMC; *n* = 14) from a tertiary care hospital in South India. Differential cytokine expression analysis (minimum log2-fold difference; adj *P*-value < 0.05) identified bacterial PAMPs induced higher concentrations of IL-1β, IL-10, TNF-α in adults versus IL-8, GM-CSF, IFN-γ, and IL-2 in CB. CB responded to poly I:C and SARS-CoV-2 lysate with a dominant IL-8 response, whereas in PBMC, CXCL-10 dominated poly I:C, but not SARS-CoV-2, responses, highlighting potential IL-8 importance, in the absence of Type I Interferons, in antiviral CB immunity. *Candida albicans* was the only PAMP to uniformly induce higher secretion of effectors in CB. The predominant source of IL-8/IL-6/TNF-α/IL-1β in both CB and PBMC was polyfunctional monocytes and IFN-γ/IL-2/IL-17 from innate lymphocytes. Correlation matrix analyses revealed IL-8 to be the most differentially regulated, correlating positively in CB versus negatively in PBMC with IL-6, GM-CSF, IFN-γ, IL-2, consistent with more negatively regulated cytokine modules in adults, potentially linked to higher anti-inflammatory IL-10. Cord and adult blood from India respond robustly to PAMPs with unique effector combinations. These data provide a strong foundation to monitor, explore, mechanisms that regulate such immunity during the life course, an area of significant global health importance given infection-related infant mortality incidence.

## Teaser

Immune profiling analysis was conducted on umbilical cord blood (CB; n=21) and adult peripheral blood mononuclear cells (PBMC; n=14) from India. Six pathogen-associated molecular patterns (PAMPs) were used to stimulate immune responses. Differential secreted cytokine expression analysis, relative to no stimulation control, as measured by a multiplex ELISA, revealed PAMP-specific distinct effectors patterns. The predominant cellular source of IL-8/IL-6/TNF-a/IL-1ÃŸ in both CB and PBMC, as determined by intracellular cytokine staining, was polyfunctional monocytes and IFN-? /IL-2/IL-17/IL-10 from innate lymphocytes.

## Graphical Abstract

**Graphical Abstract:**
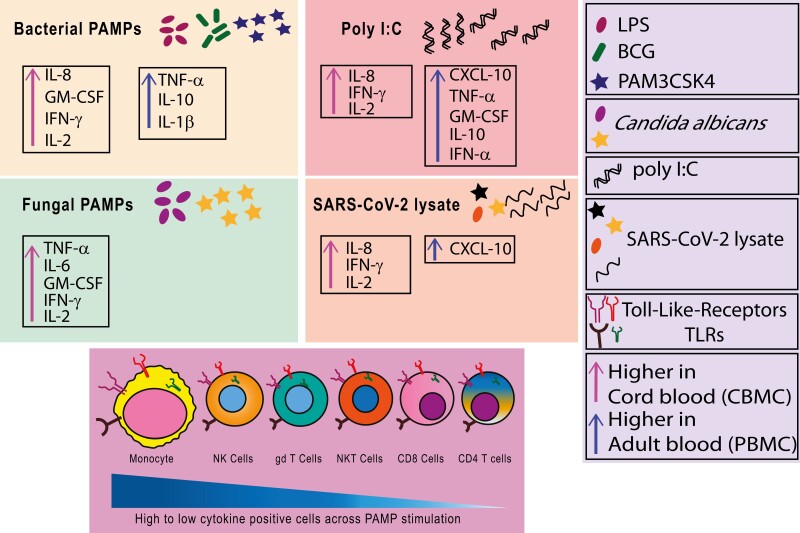

## Introduction

Neonatal mortality is a significant contributor to the overall mortality rate of children under the age of five worldwide [1]. This issue is particularly pronounced in low- and middle-income countries. In India, the neonatal mortality rate (1.9%) significantly contributes to the country’s infant mortality rate (2.5%) [2]. Eighteen per cent of neonates born in India have a low birth weight [3]. The major causes of neonatal deaths are prematurity and low birth weight (46.1%), birth asphyxia and birth trauma (13.5%), and neonatal pneumonia (11.3%) [4]. The immune system in neonates differs quantitatively and qualitatively from the adult immune system, potentially leading to increased vulnerability to infections compared to adults [5–7]. Newborn babies heavily depend on their innate immune systems for protection due to known deficiencies in neonatal adaptive immunity linked to limited priming of the immune system *in utero* [5]. Numerous studies have investigated cellular functions to identify the differences between neonatal and adult immune systems. Notably, human newborn monocytes/macrophages and dendritic cells have been found to display distinct cytokine profiles compared to their adult counterparts [8–10] with notable disparities in cellular functional responses between neonates and adults. When exposed to most TLR ligands, neonatal innate immune cells tend to produce lower levels of IL-12p70 and IFN-γ. Neonatal cells secrete moderately diminished TNF-α production but comparable or even elevated levels of IL-1β, IL-6, IL-23, and IL-10 compared to their adult counterparts [11, 12].

Although the critical importance of newborn innate immunity is universally accepted, it is worth noting that several studies highlight variations in cytokine effector responses to pathogen-associated molecular pattern (PAMPs) stimulation in infant samples [13, 14] and cord blood [15, 16] collected from different countries, reflecting inherent population variations in newborn innate immunity that may be linked to ethnicity, geography, nutritional status of mother, and importantly environmental cues to which the mother is exposed. In this context, understanding of the breadth of cord blood innate immunity in India is extremely limited with only a few publications recording frequencies of major immune cell lineages in full-term and umbilical cord blood and its comparison with adults [17–19] with no reports, to date, of the functional status of innate immune responses. The objective of our work was to address this knowledge gap and to assess cord blood innate effector function to a broad range of PAMPs, not previously tested, including bacterial (LPS, BCG, and Pam3CSK4), fungal (heat-killed *Candida albicans*), viral (poly I:C and SARS-CoV-2 lysate) PAMPs tested on umbilical cord blood mononuclear cells (CBMCs) collected from a hospital in South India.

We provide the first comprehensive analysis of the concentrations and coordinated expression patterns of 10 effectors secreted across bacterial, fungal, and viral PAMPs in CB versus adult PBMC in India placed in the context of their cellular source. Our data demonstrate that CB cells collected from mothers registered in a South Indian hospital have robust innate immune responses, crucial for combating recognized bacterial, fungal, and viral pathogens.

## Methods

### Mother and infant dyad

Apparent healthy pregnant women attending routine antenatal care at the outpatient unit of the Obstetrics and Gynaecology department of St. John’s Medical College Hospital were invited for the study. A consort flowchart showing the details of the recruitment and study protocol has been provided in Supplementary Fig. S1. Based on the inclusion criteria (pregnant women willing to consent to the study protocol, willing for a blood draw during pregnancy, collection of cord blood at delivery, women with term delivery and willing to continue postnatal follow-up with serial blood collection of the infant after birth, 3rd month and at 1 year) were enrolled in the 3rd trimester of pregnancy (>32 weeks of gestation until delivery). Women with a prior history of tuberculosis or *any* contact with TB-infected individuals, with a history of allergy to BCG or on immunosuppressants or were immunosuppressed (through clinical diagnosis) and those moving out of the study site before delivery were excluded from the study. At recruitment, a short validated questionnaire was administered to obtain information on their demographic and socio-economic status, basic anthropometry at screening (weight in kg; height in cm), and a brief history of illness during the previous month of pregnancy if any was recorded. Information regarding the tuberculosis infection (past history or current status) in the family members/associates with whom the subject resided was also documented. A total of 102 subjects were recruited. None of the mothers were undernourished (BMI < 18.5 kg/m^2^) or severely anaemic (haemoglobin concentration < 7.0 g/dl) at recruitment. However, 52.4% were mild-to-moderate anaemic in the third trimester of pregnancy. Approximately 76% of the women were pregnant for the first time. Almost ~60% of the subjects were excluded from the study due to preterm births or those who missed the cord blood collection due to delivery at odd hours of the day or had emergency delivery due to medical complications or moved out of the study hospital for delivery. The cord blood for 41 subjects was collected at delivery and had term infants. Of these 41 infants, two were born preterm while 4 infants were born with low birth weight (bwt < 2500 g). For the analysis, the cord blood with a sample size of *n* = 21 exhibited good viability (> 70%), which was solely analyzed and utilized for this study. Of the 21 mothers, all had term infants and none of the babies were born with low birth weight. The mean birth weight of the infants was 3038 g. This cohort comprised 67% of male infants and 33% were born female. The details of only these maternal and infant characteristic summaries are in Tables 1 and 2, respectively.

**Table 1.** Maternal characteristics at recruitment

Parameters (*n* = 21)	Mean ± SD	Range (Min, Max)	Median (IQR)
Age (years)	25.2 ± 3.8	20.0, 33.0	25.0 (22.0, 28.0)
Gestational age at recruitment (weeks)	35.8 ± 2.8	28.6, 39.4	36.0 (33.9, 38.1)
Education^**δ**^			
<High school		6 (28.6)	
PUC + diploma		11 (52.4)	
University and above		4 (19.0)	
Parity^**δ**^			
Nulliparous		16 (76.2)	
Multiparous		5 (23.8)	
Blood pressure			
Systolic blood pressure (mmHg)	117 ± 5.3	110, 130	120 (110, 120)
Diastolic blood pressure (mmHg)	77 ± 4.6	70, 86	80 (76, 80)
Height (cm)	155.8 ± 5.6	142.0, 166.0	158.0 (153.0, 159.0)
Weight (kg)	69.2 ± 7.8	57.0, 84.0	70.9 (64.0, 74.5)
BMI (kg/m^2^) Normal (18.5–22.9 kg/m^2^) Overweight (23–24.9 kg/m^2^) Pre-obese (25–29.9 kg/m^2^) Obese (≥30.0 kg/m^2^)	28.6 ± 3.5 2 (9.5%) 1 (4.8%) 11 (52.4%) 7 (33.3%)	21.5, 36.1	28.5 (26.8, 30.5)
Hemoglobin (g/dl) Mild–moderate anaemic (7.1–11.9 g/dl) Normal (>12 g/dl)	11.9 ± 0.9 11 (52.4%) 10 (47.6%)	10.5, 14.0	11.9 (11.2, 12.4)

None of the subjects were undernourished, i.e. (BMI < 18.5 kg/m^2^) or were severe anaemic.

Data are presented in mean ± SD; Min, Max; median (Q1, Q3); *N* (%) for categorical data.

^**δ**^Values represent number (percentages).

**Table 2.** Infant characteristics at birth

Parameters (*n* = 21)	Mean ± SD	Range (Min, Max)	Median (IQR)
Gestational age at delivery (weeks)	39.3 ± 0.8	37.9, 40.6	39.4 (38.5,40.0)
*Type of delivery* ^***δ***^			
Normal vaginal delivery	11 (52%)		
Caesarean section delivery	10 (48%)		
*Gender* ^***δ***^			
Male	14 (67%)		
Female	7 (33%)		
Birth weight (g)^**φ**^ Low birth weight (bwt < 2500 g)	3038.3 ± 239.7 0 (0.0 %)	2650.0, 3512.0	3053.0 (2880.0, 3210.0)
Length at birth (cm)	49.6 ± 0.8	48.3, 51.1	49.5 (49.2, 50.0)

Data are presented in mean ± SD; Min, Max; median (Q1, Q3).

^**δ**^Number (percentages).

### Clinical recruitment of adults for this study

The adult study was conducted among apparent healthy volunteers of both genders within the age group of 18-25 years old. On the day of screening; day T0, the volunteers had a short validated questionnaire administered that included information on socio-demography and other relevant clinical details such as history of their previous BCG immunization and the vaccination at birth was verified through medical/ immunization records or verbal information or by the presence of the BCG scar at the vaccine site. Anthropometric measurements were taken, including weight (kg), height (cm), and body mass index (BMI; calculated in kg/m²).. The subjects were excluded if they had at least one of the exclusion criteria similar to the list mentioned in the mother and infant study. The venous blood sample (20 ml) was drawn at recruitment (day T0) in sodium heparin tubes, processed and stored accordingly until analysis. Only baseline samples from this cohort were used in this study. A consort flowchart showing the details of the recruitment and study protocol has been provided in Supplementary Fig. S1. The detailed study protocol is described in Ref. [20]. The adult samples collected during the cord blood collection time frame and those with good viability were only used for this study.

### Cord blood and adult blood mononuclear cell isolation

Cord blood *n* = 21 (4–5 ml) and healthy adult blood *n* = 14 (18–20 ml) were collected in Na-heparin tubes (BD, Franklin Lakes, NJ, USA) and peripheral blood mononuclear cells (PBMCs) or CBMCs were isolated using 15 ml blood separation tubes (SPL Life Sciences) by density centrifugation. Briefly, blood was diluted two-fold with PBS (Gibco by Life Technologies, Washington, DC, USA) +2% FBS (Gibco), pipetted into blood separation tubes pre-filled with Lymphoprep™ (STEMCELL Technologies) and centrifuged at 1000*g* for 15 min at room temperature without deceleration. PBMCs or CBMCs from the buffy coat were washed twice with PBS + 2% FBS, then re-suspended at 10 × 10^6^ cells/ml in cryopreservation medium (90% FBS and 10% DMSO-Sigma) and incubated overnight at −80°C (in Mr Frosty™ freezing container; Nalgene, Rochester, New York, USA) and were stored in liquid nitrogen until further analyses.

### PAMP stimulation of CBMC and adult PBMC

Cryopreserved CBMC or adult PBMC were taken out in a liquid nitrogen bath. They were thawed in a 37°C water bath till a pea-sized lump of frozen material remained in the vial. One millilitre of pre-warmed RPMI (Gibco by Life Technologies) + 10% FBS + antibiotics (Gibco by Life Technologies) was added to the vials and the total contents were transferred drop by drop to a 50-ml tube containing 20 ml of warm complete media {RPMI-1640 (1×) + GlutaMAX™-1 + 25 mM HEPES [Gibco by Life Technologies] supplemented with 10% FBS [Thermo Fisher Scientific], 100 U/mL penicillin and 100 μg/mL streptomycin}. Cells were pelleted down at 800*g* for 5 min and then subjected to another wash with complete media.

Then the cells were seeded in 96-well Flat-bottom plates (Costar) at 0.4 × 10^6^ cells/well in 100 µl of complete RPMI medium. After 2 h of rest, cells were stimulated for 24 h at 37°C with 10^6^ cfu/ml of heat-killed *C. albicans* Strain SC5314 (kind gift from David Moyes; King’s College London) or 0.2 × 10^6^ cfu/ml of BCG (TUBERVAC™, Serum Institute of India) or 1 ng/ml LPS (Sigma) or 50 μg/ml Pam3CSK4 (Sigma) or 100 µg/ml poly I:C (Invivogen) or 1 µg/ml SARS-CoV-2 lysate (Native Antigen) prepared in 100 μl of RPMI medium with Glutamax and antibiotics. Cells cultured with medium alone, no stimulus, served as a negative control. Twenty-four hours later plates were centrifuged at 800*g* for 3 min and culture supernatants were collected and frozen at −80°C till ELISA was performed.

### Multiplex bead array

A customized multiplex bead array panel (R&D Systems) was used for measuring levels of TNF-α, IL-1β, IL-6, CXCL-8, GM-CSF, IL-10, IL-17, CXCL-10, IL-2, IL-13, IL-15, IL-5, IFN-γ, and IFN-α according to manufacturer’s instructions. Briefly, 50 µl of culture supernatant was incubated with 50 µl of capture magnetic beads for 2 h on a plate shaker. The magnetic beads were washed 3×. Next, beads were incubated for 1 h with a biotin-conjugated detection antibody cocktail, washed 3×, and finally incubated with Streptavidin-PE for 30 min. At the end of incubation, beads were washed thoroughly 3×, resuspended, and the samples were run on a Luminex MAGPIX xMAP plate reader. A nine-point standard curve was included in each run and cytokine/chemokine concentrations were calculated using the xPONENT™ software. The spontaneous cytokine release in cells cultured with medium alone was subtracted from all PAMP stimulation conditions.

### CBMC and adult PBMC ICS assay

Innate immune responses were tracked as previously described [21]. Briefly, cryopreserved CBMC were thawed, and seeded in 96-well round-bottom plates (Costar) at 1 × 10^6^ cells/well in complete RPMI medium {RPMI-1640 (1×) + GlutaMAX™-1 + 25 mM HEPES [Invitrogen] supplemented with 10% FBS [Thermo Fisher Scientific], 100 U/ml penicillin and 100 μg/ml streptomycin}. After 2 h of rest, cells were stimulated for 6 h at 37°C with BCG, LPS, *C. albicans*, poly I:C and SARS-CoV-2 lysate at a final concentration of 0.2 × 10^6^ cfu/ml, 1 ng/ml, 10^6^ cfu/ml, 100 µg/ml, and 1 µg/ml, respectively. For negative control, cells were incubated with an equivalent volume of sterile water. Brefeldin A (1×, BioLegend) was included in the last 4 h to prevent cytokine release. After 6 h, cells were stained first with viability dye AviD followed by staining with 50 µl cell surface (CS) staining cocktail prepared in PBS + 2% FBS for 30 min at room temperature (RT) in the dark. Next, cells were washed with PBS and fixed with 1× FACS lysis buffer (BD Biosciences, Cat. No.349202) for 10 min at RT. After washing, cells were permeabilized by adding 200 μl 1× Perm/Wash solution (BD Biosciences) and incubated at RT for 20 min. Pelleted cells were immediately stained with a 50-µl cocktail containing antibodies against intracellular (IC) markers for 30 min at RT. Finally, cells were washed and re-suspended in 100 μl of 1% paraformaldehyde (Electron Microscopy Sciences, Hatfield, PA, USA) for flow cytometry analysis (see Supplementary Tables S1 and Table S2 for details on the flow cytometry antibody panel used).

### Correlation analysis

Correlation analysis of stimulant-cytokine pairs was done using Spearman’s rank correlation method implemented in the *rcorr* function of *Hmisc* package of R statistical programming language. Obtained outputs were fed to *corrplot*, another R package with same function name, to generate correlation plot with a default colour scheme for negative- and positive-correlations. Also, *P* values of correlation significance were corrected for multiple testing using the Benjamini–Hochberg (BH) method. The final correlation plot was enabled to depict levels of significance (i.e. *P < 0.05, **P < 0.01, ***P < 0.001) between correlating parameters in respective glyphs, and glyph’s borders are highlighted in magenta colour if it falls below the threshold of adjusted *P*–value, i.e. Padj < 0.05. Graphpad Prism software was used for principal component analysis (PCA) and plotting of cytokines response to stimulations.

### Differential cytokine responses

Adult versus cord blood differential cytokine responses to selected PAMPs were determined using linear model implemented in *limma* package of R with adjusted *P*-value < 0.05 (Benjamini–Hochberg) and absolute fold change > 2. Data were log2 transformed before differential analysis. Volcano plots were drawn in R.

### Flow cytometry data analysis

Cell fluorescence was acquired on the 5-laser, 18-parameter BD FACSAria™ Fusion flow cytometer (BD Biosciences, San Jose, CA) using BD FACSDiva™ version 8.0.1 software. Samples were analyzed using FlowJo 10.8.0 (BD Biosciences). Briefly, cells were first gated on FSC-H vs FSC-A to exclude doublets and then on the singlet gate AviD positive or dead cells (FSC-A vs AviD) were gated out. The gated AviD negative cells were carried forward for further analysis. All cytokine frequencies are reported after background subtraction of identical gates applied on matched negative controls. The assay cut-off was set at ≥ 0.01%. Background subtractions were performed in Pestle version 1.8. The polyfunctionality of Monocytes and NK cells expressing combinations of IL-1β, IL-6, IL-8, and TNF-α was analyzed with SPICE version 6.1 software [22] as described previously [20].

### Statistical analysis

Data were analyzed using FlowJo 10.9.0. Graphical representations were performed in Prism version 10.0.0 (GraphPad, San Diego, CA, USA). Statistical tests were performed in Prism. Nonparametric tests were used for all comparisons. Mann–Whitney *U* or Wilcoxon signed-rank *t*-tests were applied for unpaired or paired comparisons.

## Results

### PAMP-induced cytokine responses differ in magnitude and effector combinations between CBMCs and adult PBMCs

The potency and breadth of cord and adult blood responses were tested on a panel of bacterial, fungal, and viral pathogen-associated molecular patterns (PAMPs), including BCG (TLR2, TLR4, and TLR9 agonist) [23–25], LPS (Gram-negative bacteria, TLR-2/TLR-4-MD-2 agonist) [26, 27], Pam3CSK4 (TLR-2agonist) [28, 29] heat-killed *C. albicans* [30] (fungal, TLR2, TLR4 agonist), viral RNA mimic poly I:C (TLR-3 agonist) [31], and SARS-CoV-2 lysate [32]. A panel of 10 effector cytokines was measured in culture supernatant harvested at 24 h post-stimulation, including IL-6, IFN-α, IL-1β, TNF-α, IFN-γ, IL-2, IL-8, CXCL-10, GM-CSF, and anti-inflammatory/immune-modulatory IL-10. Supplementary Fig. S2 provides background concentrations of all analytes. IL-8, TNF-α, and IL-6 were the most abundant effectors that were spontaneously secreted across many donors; CB cells secreted significantly higher concentrations of TNF-α and IL-2, whereas adults spontaneously secreted higher concentrations of IL-10 and CXCL-10 compared to CB.

**Figure 1.**
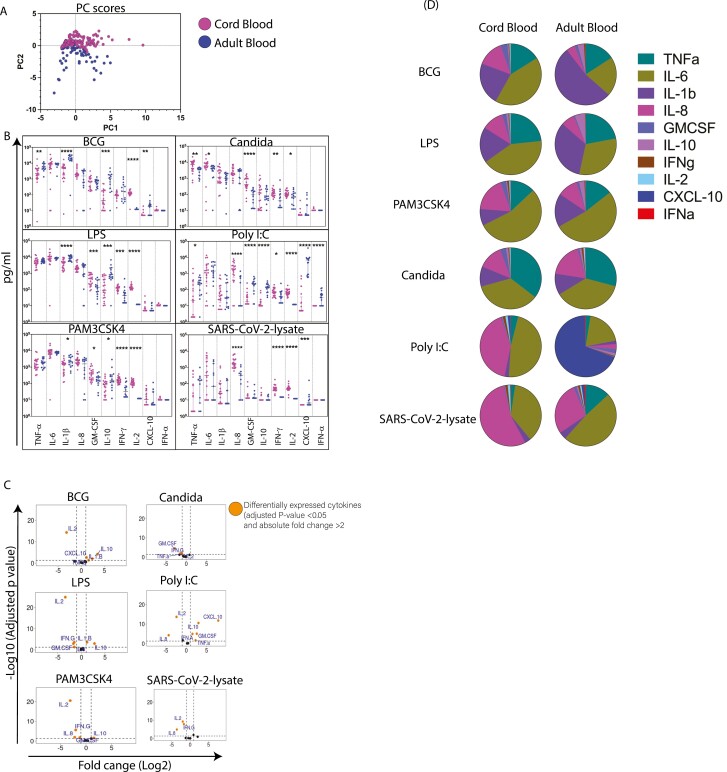
Magnitude of PAMP-induced cytokine responses differ between cord blood (CB) and adult peripheral blood mononuclear cells (PBMC). Mononuclear cells isolated from infant cord (*n* = 21) and adult blood (*n* = 14) were stimulated with multiple PAMPs for 24 h after which supernatants were collected and cytokines were measured by a customized cytokine bead array to study expression of 10 key innate and adaptive analytes. No-stimulation cells were used as negative controls and subtracted from all the PAMP-stimulated samples. (A) Principal component analysis (PCA) of adult and cord blood multiplex dataset including 10 cytokines and 6 stimulations. Principal components of the immune parameters measured in adults and cord blood. Each data point on the plot represents an individual observation in the dataset. (B) The absolute concentrations of analytes in adult and cord blood for bacterial (BCG, LPS, and Pam3CSK4), fungal (*Candida albicans*) PAMPs and viral PAMPs (poly I:C and SARS-CoV-2 lysate). (C) Adult versus cord blood differential cytokine responses to selected PAMPs are shown in volcano plots. Differentially expressed cytokines (adjusted *P*-value < 0.05 and absolute fold change > 2) are labelled. Over-expressed cytokines in adult are shown in right compartment (right side to the vertical dotted line) while left compartment have over-expressed cytokines in cord blood group. (D) Pie chart showing the contribution of each cytokine for bacterial (BCG, LPS, and Pam3CSK4), fungal (*C. albicans*), and viral (poly I:C and SARS-CoV-2 lysate) PAMPs in cord blood and adults. Mann–Whitney test was used for comparisons shown in (B) *P* < 0.05 was considered significant. *P ≤ 0.05, **P < 0.01, ***P ≤ 0.001, ****P ≤ 0.0001.

Two-dimensional PCA of background subtracted cytokine concentrations from the 6 stimulation conditions, 10 effectors, and 2 groups (CB versus PBMC) in Fig. 1A highlights the two groups to clearly segregate, indicating major differences between adult and cord blood overall cytokine expression profiles with clear segregation of most, but not all, subjects. Scatter plot analysis of the same data (Fig. 1B) reveals the spread of secreted cytokine concentrations within a group and differences between CB and adults to be both effector and PAMP specific (Fig. 1B). Volcano plots in Fig. 1C giving adjusted *P*-values with log-fold changes.

PAMP responses that were significantly higher in adults (Fig. 1C, right of vertical line) included: IL-1β (2-4 fold), IL-10 (2-24 fold), and TNF-α (3-fold in BCG) to bacterial PAMPs (BCG, LPS, and Pam3CSK4).

PAMP responses that were significantly higher in CB (Fig. 1C, left of vertical line) included: IL-8, GM-CSF, IFN-γ, and IL-2 to bacterial PAMPs (LPS, Pam3CSK4), TNF-α, GM-CSF, IFN-γ, IL-2 for the fungal PAMP, and IFN-γ, IL-2 and IL-8, being higher in CB cultures to viral PAMPs: poly I:C and SARS-CoV-2 lysate (Fig. 1C). These data highlight that the magnitude of effector responses and effector combinations inherently differ between CB and PBMC in a PAMP-specific manner. Interestingly, *C. albican*s was the only PAMP which uniformly induced higher secretion of effectors in CB (Fig. 1C), indicating that CB cells may be particularly sensitive to this PAMP.

Further analysis was undertaken to assess the proportion of a given effector secreted to each stimulus and represented in PIE CHARTS (Fig. 1D), enabling dominant and minor effector combinations to a given PAMP to be visually identified. Whilst the scatter plots in Fig. 1B show significant differences in the magnitude of effectors between CB and adult cells to LPS, Pam3CSK4 and *C. albicans* (see also Fig. 1C), the PIE charts in Fig. 1D show overall similarity of effector combinations to the same three PAMPs, indicating that whilst concentrations may differ, both CB and adults respond by secreting similar combinations of effectors to LPS, Pam3CSK4, and *C. albicans*. However, responses to BCG were distinct, with IL-1β (purple) dominating in adults compared to IL-6 (olive green) in CB. The most striking difference was to the viral PAMPs: poly I:C predominantly induced IL-8 (magenta-41%) in CB versus CXCL-10 (dark blue- 69%) in adults; SARS-CoV-2 lysate induced IL-8 (54%) in CB versus IL-6 (olive green, 49%) in adults. Taken together, data in Fig. 1 confirm that cord and adult blood from India respond robustly to PAMPs by secreting unique combinations of effectors.

### Correlation matrix analysis highlights more negatively regulated cytokine modules in adults compared to CB

**Figure 2.**
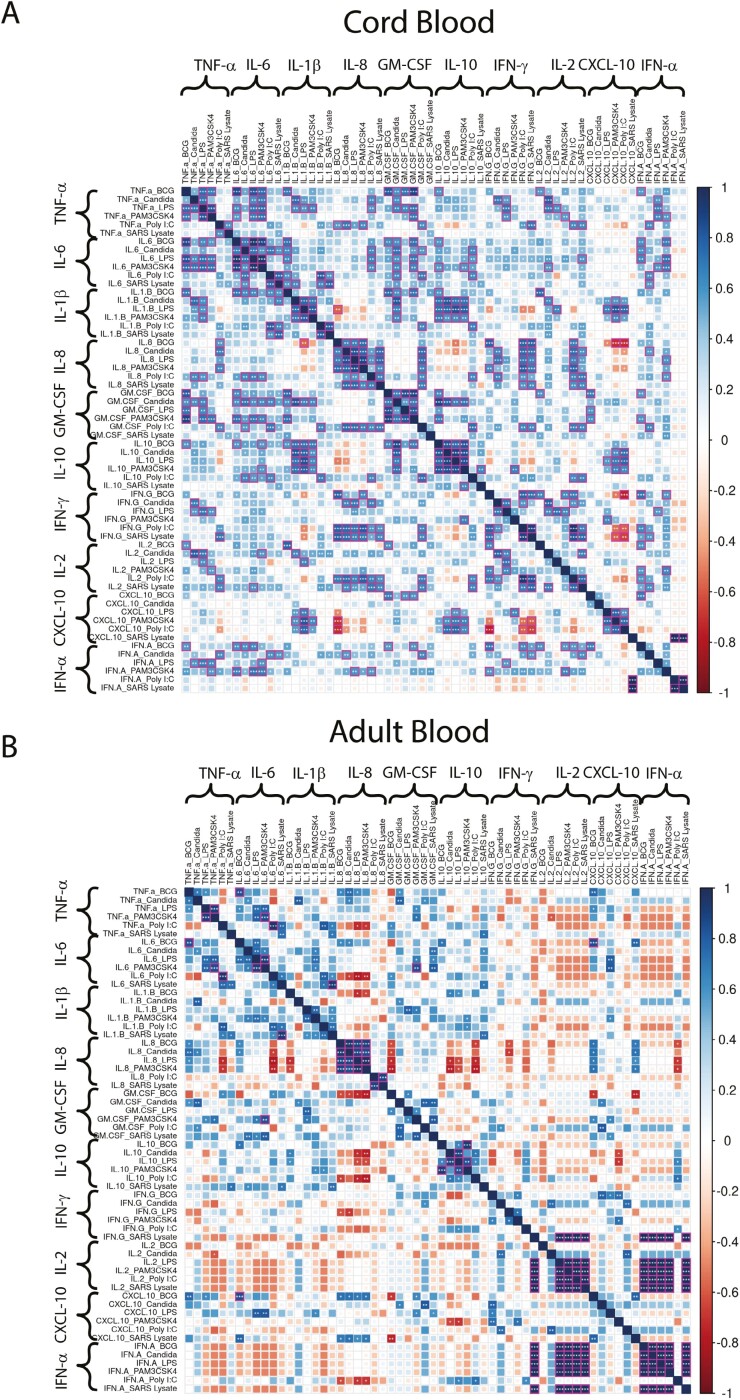

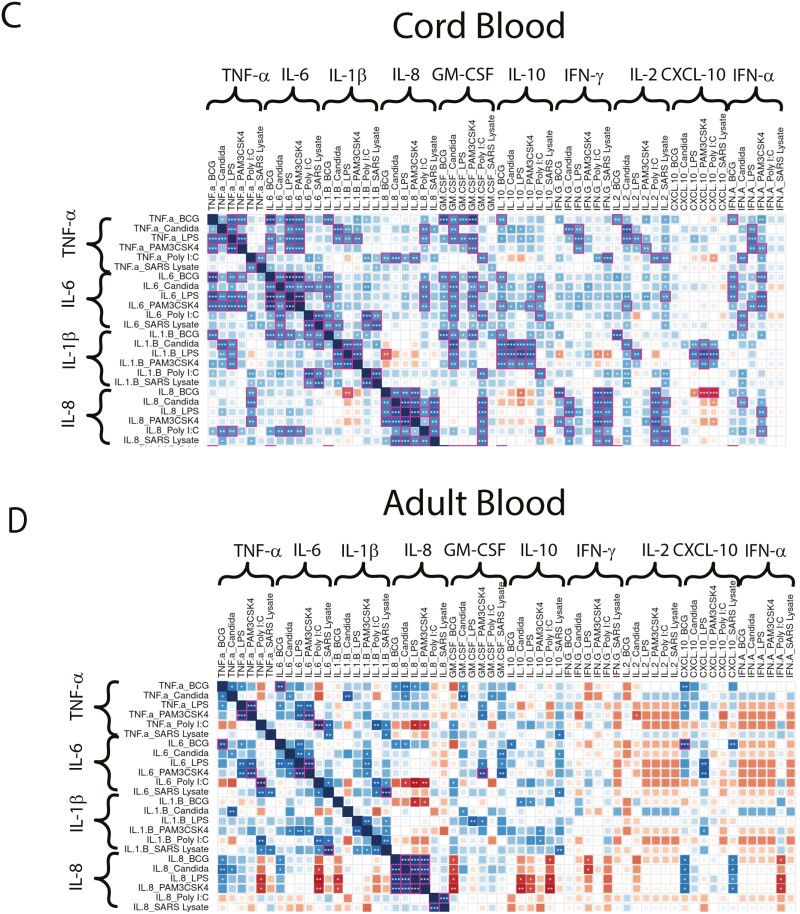
Coordinated expression patterns of PAMP-induced cytokine modules differ markedly between CB and PBMC. Correlation matrices were generated from multiplex data shown in the figure for cord and adult peripheral mononuclear cell responses. Spearman’s correlation method is used for the clustering of cytokine-stimulant pairs. (A) Correlation of all cytokines measured in cord blood (CBMC) (*n* = 21) across all the PAMPs tested. (B) Correlation of all cytokines measured in adult blood (PBMC) (*n* = 14) across all the PAMPs tested. (C) Correlation of IL-1β, TNF-α, IL-8, and IL-6 in cord blood (CBMC) across all the PAMPs tested. (D) Correlation of IL-1β, TNF-α, IL-8, and IL-6 in adult blood (PBMC) across all the PAMPs tested. Spearman’s rank correlation was used. *P*-values of correlation significance were corrected for multiple testing using the Benjamini–Hochberg (BH) method. *P* < 0.05 was considered significant. *P ≤ 0.05, **P < 0.01, ***P ≤ 0.001, ****P ≤ 0.0001.

Correlation matrices provide insights into how multiple effectors may be co-expressed, revealing cytokine modules that are either positively (blue) or negatively (red) correlated with the potential to provide mechanistic insights into differences in adult versus CB PAMP responses. Secreted cytokine concentrations shown in Fig. 1 were therefore subjected to correlation analysis and grouped in such a way as to reveal correlation patterns of each effector across multiple PAMPs. This bird’s eye view (Fig. 2A and B) highlighted effectors that were positively correlated across multiple PAMPs in both CB and PBMC (groups of blue squares with magenta edges with stars). Importantly, this analysis highlighted fewer negatively correlated modules in CB (fewer red boxes, Fig. 2A) compared to adult PBMC (Fig. 2B), indicating a trend for a higher level of controlled expression of multiple effectors in adults compared to CB. A more focused analysis of key differences between CB and PBMC is illustrated in Fig. 2C and D. Three key innate pro-inflammatory mediators TNF-α, IL-1β, and IL-6, critical for innate immunity, positively correlated with each other (overall pattern of blue boxes), with many of the correlations reaching significance (blue box, magenta edges with star) in CB (Fig. 2A and C) but not the adult (Fig. 2B and D). This difference is consistent with higher expression of IL-10, a negative regulator of pro-inflammatory cytokines in adults (see Fig. 1B and C), likely resulting in more controlled expression of adult TNF-α, IL-1β, and IL-6 expression. Another effector module that differed significantly between cord and adult blood, was IL-8. CB cells displayed positive correlations of IL-8 with TNF-α, IL-6, GM-CSF, IFN-γ, and IL-2 and negative correlations with IL-10 and CXCL-10 (Fig. 2C), whereas in adults, IL-8 exhibited negative correlations with all cytokines (including IL-10 where a strong negative correlation was noted) except for TNF-α and CXCL-10 (Fig. 2D). This is consistent with more dominant IL-8 secretion in CB compared to adult cultures due to IL-8 concentrations increasing concomitantly with other pro-inflammatory effectors: TNF-α, IL-1β, IL-6, GM-CSF, IL-2, and IFN-γ. The negative correlation of IL-8 and IL-10 in both the CB and adult correlation matrix indicates that IL-8 expression is moderated by IL-10, and this is particularly strong in adults. This analysis further confirms (i) PAMP effector responses to be unique to CB and adult, (ii) some effectors to be co-ordinately expressed across multiple PAMPs, and (iii) of these coordinated modules, the IL-8 module to be one of the most markedly different between CB and adult.

### Polyfunctional monocytes are a major cellular source of innate cytokines and chemokines in both CBMC and PBMC

We next determined if the differential effector secretion patterns described above are due to differences in CB versus PBMC cellular responses. The cellular source of cytokines and chemokines to PAMP stimulation was therefore assessed using an advanced 18-colour flow-cytometry staining panel (Supplementary Table S1), which included lineage markers for monocytes, NK cells, NK T cells, γδ T cells, and adaptive subsets: CD4 and CD8 T cells. Three PAMPs that were markedly immunostimulatory (Fig. 1B) were first included: BCG, LPS, and *C. albicans*. The effectors measured were IL-8, IL-6, TNF-α and IL-1β, IFN-γ, IL-2, IL-17, and IL-10. Supplementary Figs. S3 and S4 show an optimized flow cytometry gating strategy and representative staining, respectively.

**Figure 3.**
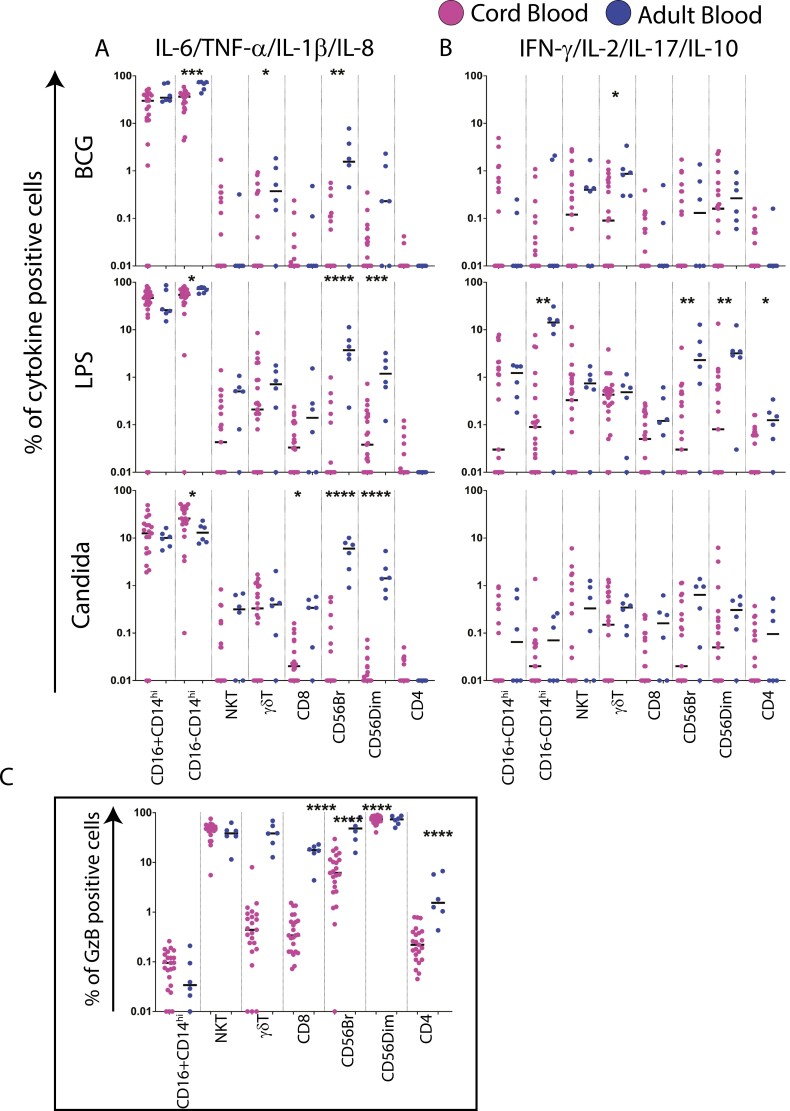
Monocytes were the major cellular source of cytokines and chemokines in CBMC and PBMC. CBMC (*n* = 21) and adult PBMCs (*n* = 6) were stimulated with 0.25 × 10^6^ cfu/ml BCG or 1 ng/ml LPS or 10^6^ cfu/ml *Candida albicans* for 6 h after which cells were stained with a panel of antibodies to study the expression of IL-1β, IL-8, IL-6, TNF-α, IL-17, IL-10, IFN-γ, and IL-2. No-stimulation cells were used as negative controls and subtracted from all the PAMP-stimulated samples. (A) Frequency of immune cell subsets producing at least one of IL-6, TNF-α, IL-1β, and IL-8 cytokines in BCG, LPS, and *C. albicans* in cord blood (CBMC) and adult blood (PBMC) across immune cell subsets. (B) Frequency of immune cell subsets producing at least one of IFN-γ, IL-2, IL-17, and IL-10 cytokines in BCG, LPS, and *C. albicans* in cord blood (CBMC) and adult blood (PBMC) across immune cell subsets. (C) Frequency of immune cell subsets producing Granzyme B in No-stimulation control. Mann–Whitney test was used for comparisons shown in (B and C) *P* < 0.05 was considered significant. *P ≤ 0.05, **P < 0.01, ***P ≤ 0.001, ****P ≤ 0.0001.

Cytokine-expressing cell frequencies were analyzed based on the abundance of expression to these bacterial fungal PAMPS, as two groups: cell lineages expressing IL-8 and/or IL-6, TNF-α, and IL-1β were abundant and grouped, with median frequencies of cells expressing these cytokines (after background subtraction) ranging from 0.01% to 73% (Fig. 3A). Cell lineages expressing IFN-γ and/or IL-2, IL-17, and IL-10 were lower and analyzed as another group, with median frequencies of cells expressing these cytokines (after background subtraction) ranging from 0.01% to 14% (Fig. 3B). Supplementary Fig. S5 shows the background frequencies of cytokine-positive cells; monocytes (CD16 + CD14^high^ and CD16-CD14^high^) from many donors expressed the more abundant cytokines in the absence of stimulation.

For high-expressing cytokines (IL-8, IL-6, TNF-α, and IL-1β), monocytes were identified as the predominant cellular source (Fig. 3A); in contrast, a broad range of cell lineages including monocytes, NK T cells, γδ T cells, CD56bright, CD56Dim NK cells, CD4, and CD8 T cells expressed IFN-γ, IL-2, IL-17, and IL-10 (low expressing cytokines) (Fig. 3B); this overall expression pattern and hierarchy (monocytes > NK ≥ γδ T ≥ NKT ≥ CD8 ≥ CD4) was common to both CB and adults. However, differences in the magnitude of some cellular subsets were noted. Thus, CD16-CD14^high^ cytokine-secreting monocyte frequencies were significantly higher in adults compared to CB to BCG and LPS stimulation, consistent with higher TNF-α and IL-1β secretion to the same PAMPs in the cytokine secretion assay by PBMC (Fig. 1C). However, the strikingly higher secretion of IFN-γ and IL-2 in CB to BCG, LPS, and Candida in the cytokine secretion assay (Fig. 1C) could only be partly explained by cell frequencies as adults had higher frequencies of IFN-γ+ IL-2 + NK T cells, γδ T cells, CD56Dim NK cells, CD4, and CD8 T cells to BCG but not LPS; indeed, the frequencies of these lineages was overall higher in adults (Fig. 3). These differences between the adults and CB were confirmed when individual cytokine frequencies were analyzed in the context of cell lineages confirming monocytes to be the dominant effectors in both adult and cord blood for all three PAMPs in the context of all effectors tested (Supplementary Fig. S6). The cellular source of Granzyme B was analyzed separately as a key mediator of both innate and adaptive cytolytic effector function (Fig. 3C). Consistent with the known cytolytic function of this mediator, NKT and CD56dim cells were the highest expressing cells in both adult and CB (47% NKT, 74% CD56dim in CB versus 38% NKT, 74% CD56dim in adults) with frequencies of γδ T cells, CD56^Bright^, CD8, CD4 T Granzyme B + cells being significantly higher in adults versus CB (Fig. 3C), emphasizing the importance of Granzyme B in adult innate immunity.

**Figure 4.**
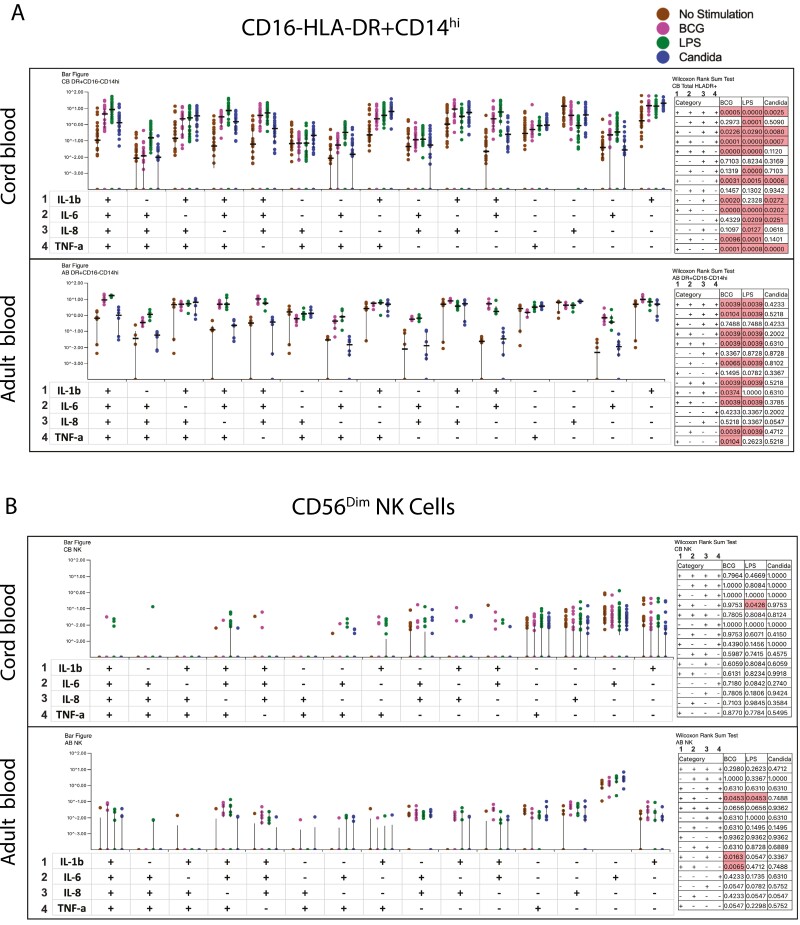
Quality of the PAMP-specific monocytes and NK cell response in cord and adult blood. CBMC (*n* = 21) and adult PBMCs (*n* = 6) were stimulated with 0.25 × 10^6^ cfu/ml BCG or 1ng/ml LPS or 10^6^ cfu/ml *C. albicans* for 6 h after which cells were stained with a panel of antibodies to study the expression of IL-1β, IL-8, IL-6, TNF-α, IL-17, IL-10, IFN-γ, and IL-2 in various immune cell subsets. No-stimulation cells were used as negative controls. Boolean gates were created from the individual cytokines (IL-1β, IL-8, IL-6, and TNF-α) in FlowJo to divide responding cells into 15 distinct subsets corresponding to all possible combinations of these functions, and the data were analyzed using SPICE software. PAMP-induced immune responses to BCG, LPS, and Candida were compared with no stimulation control. Data were analyzed for statistical significance using Wilcoxon signed-rank test. Log data analyzed in all cases. *P* < 0.05 was considered statistically significant.

Given monocytes were a dominant PAMP-responding population in both adults and CB, we assessed if this cell subset was polyfunctional in terms of simultaneously expressing multiple effectors (TNF-α, IL-1β, IL-6, and IL-8), a feature associated with more robust immune function using SPICE software [22]. Figure 4A shows the frequencies of CD16-, HLA-DR+, CD14 + monocytes expressing combinations of 4, 3, 2, and 1 effectors in response to LPS, BCG, and *C. albicans* relative to no stimulation control (red bar) with significant differences highlighted in a *P*-value table-shaded pink, calculated by SPICE software. Whilst CB showed significant induction of polyfunctional monocytes to BCG, LPS, and *C. albicans*, polyfunctional monocytes were markedly absent to *C. albicans* stimulation in adult PBMC (Fig. 4A). As adults were not only capable of responding to *C. albicans* but had higher circulating frequencies of NK effectors expressing IL-6/TNF-α/IL-1β/IL-8 compared to CB (Fig. 3A), we probed the polyfunctionality of NK cells. CD56bright cell numbers were very low to obtain meaningful data from SPICE (data not shown). Data in Fig. 4B highlight the CD56dim NK cell population to comprise only a very limited range of 2 + and 3 + subsets that were restricted to LPS and BCG, but not Candida, stimulation. These data highlight polyfunctionality to be a striking feature of monocytes, not NK cells, and adults to lack a polyfunctional monocyte and NK cellular response to *C. albicans*.

### Cellular analysis confirms the paucity of CXCL-10 response in CB to viral PAMPs

**Figure 5.**
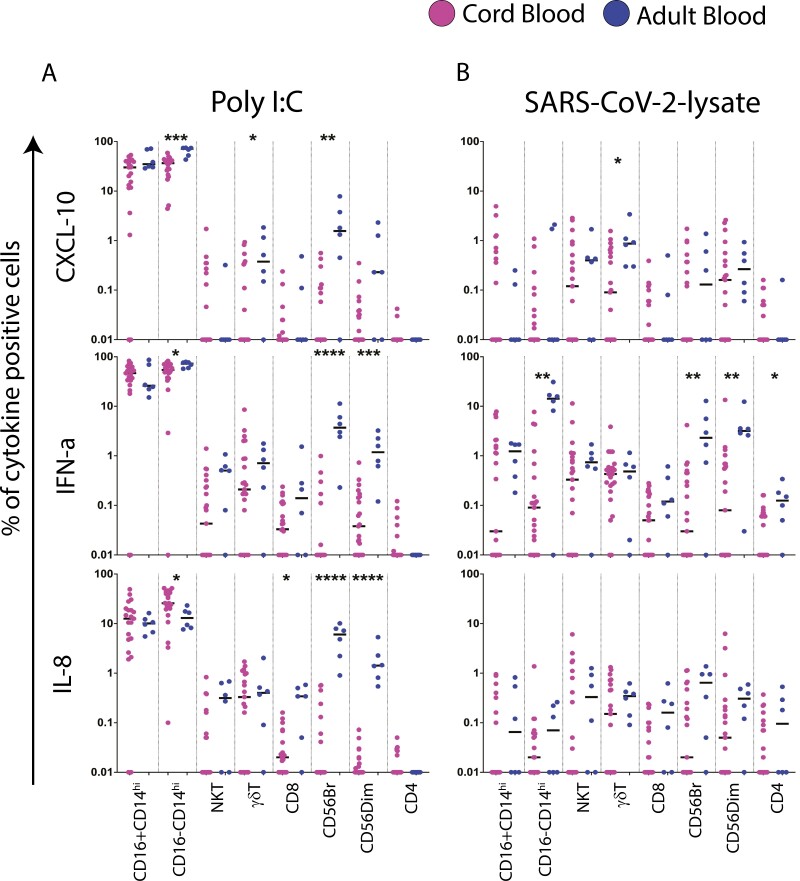
Cellular analysis confirms the paucity of CXCL-10 response in CB to viral PAMPs. CBMC (*n* = 6) and adult PBMCs (*n* = 6) were stimulated with 100 µg/ml poly I:C or 1 µg/ml SARS-CoV-2 lysate for 6 h after which cells were stained with a panel of antibodies to study the expression of IL-1β, IL-8, IL-6, TNF-α, CXCL-10, and IFN-α. No-stimulation cells were used as negative controls and subtracted from all the PAMP-stimulated samples. (A) Frequency of immune cell subsets producing CXCL-10, IFN-α, and IL-8 after being stimulated with poly I:C and SARS-CoV-2 lysate in cord blood (CBMC in pink) and adult blood (PBMC in blue). Mann–Whitney test was used for the comparisons shown. *P* < 0.05 was considered significant. *P ≤ 0.05, **P < 0.01, ***P ≤ 0.001, ****P ≤ 0.0001.

We next determined if the major differences in effector secretion patterns between CB and PBMC to the viral PAMPs (Fig. 1) were reflected at the cellular level. A bespoke 14-colour immunostaining panel was optimized (Supplementary Table S2) to probe specifically expression of CXCL-10, IFN-α, IL-8 (identified to be significantly different—Fig. 1C) as well as IL-1β, IL-6, and TNF-α with Supplementary Fig. S8A and B showing an optimized flow cytometry gating strategy and representative staining, respectively. Figure 5 and Supplementary Fig. S8 showing background subtracted cell frequencies to poly I:C and SARS-CoV-2 lysate stimulation, confirms monocytes to be the predominant cellular source of all the mediators tested in both adult and cord blood.

The data highlight (a) CXCL-10 + monocytes to be markedly lower in CB compared to PBMC to the canonical viral PAMP, poly I:C; median frequencies in CB ranged between 0.18% and 2.5% compared to PBMC where the median frequencies ranged from 24% to 46%; the data also showed SARS-CoV-2 lysate to be a poor inducer of CXCL-10 across all cell lineages confirming the cytokine secretion data and SARS-CoV-2 lysate as a poor TLR3 agonist in adults. (b) The strikingly higher secretion of IL-8 in CB to poly I:C and SARS-CoV-2 lysate in the cytokine secretion assay (volcano plot Fig. 1C) could not be explained by differential cell frequencies as CB and PBMC had similar frequencies of IL-8 + cell subsets. (c) Although the frequency of IFN-α was very low it was higher in adults compared to CB. (d) The frequency of monocytes expressing TNF-α, IL-1β, and IL-6 were lower for poly I: C and SARS-CoV-2 lysate compared to other bacterial and fungal PAMP, consistent with the cytokine secretion assay data (Supplementary Fig. S8).

## Discussion

This study provides a detailed comparative analysis of PAMP responses in mononuclear cells isolated from cord versus adult blood from South India. We highlight robust and broad PAMP-specific responses; all donors tested (CB and adult) responded by secreting one or more effectors to all PAMPs tested that could be clearly measured over and above the no-stimulation background control. To the best of our knowledge, a comprehensive comparative analysis of PAMP responses from Indian subjects assessed by 10-plex ELISA and contextualized with cellular source determined by 18-colour multiparameter flow cytometry, has not been previously reported. The one comparative study of CB versus PBMC study from India focussed on LPS-induced cytokine secretion [33] and broadly aligns with our data in terms of IL6, IL-8, and IL-1β secretion patterns; however, the markedly higher levels of IL-10 in adults that we observed were not noted in the other study [33]. Our report most closely aligns with that of Caron et al. [34], which shows markedly higher IL-10 in adults across PAMPs and a dominant IL-8 as a feature of TLR responses. Other studies across different countries have also focused largely on LPS responses [9, 35–44] and show some but not complete overlap; the most consistent observation is that IL-6, TNF-α, IL-1β, and IL-8 are the most abundant effectors of bacterial PAMPs. These data are consistent with the recognized importance of TNF-α, IL-1β, IL-6, and IL-8 as key innate immune mediators and also consistent with the emerging concept of trained immunity [45–47], which highlight the importance of TNF-α, IL-1β and IL-6 as early effectors and regulators of both innate and subsequent adaptive immune responses [21, 25]. Our data are also consistent with the recognized importance of viral PAMPs to induce the Type 1 IFN pathway [31]; thus, the highest levels of CXCL-10 (otherwise referred to as IP10) and IFN-α, two key mediators of the Type 1 IFN pathway, were induced by the viral PAMP poly I:C, rather than by bacterial/fungal PAMPs (Fig. 1B).

The cellular source intracellular staining data (Fig. 3) confirmed the same broad hierarchy of effector expression as the cytokine secretion assay: thus, frequencies of TNF-α, IL-6, IL-8, and IL-1β expressing cells were higher than cells expressing IFN-γ, IL-2, IL-17, and IL-10, a feature also noted in other reports [9, 11, 34, 48–53]. The ICS data also confirmed monocytes to be the most dominant PAMP-responding population in both adults and CB, in keeping with the recognized importance of this subset in innate immunity [11, 35–38, 40, 46, 54–58]. For the first time, we report adults to have a broader cellular PAMP response than CB with multiple lineages responding, including gamma delta, NKT, NK, and T cells, whereas monocytes and NK cells were the only two major effector populations in CB cultures (Fig. 3). The lack of cellular lineage breadth in CB may be compensated by the observation that highly polyfunctional monocytes are activated in CB to bacterial and fungal PAMP stimulation (Fig. 4), whereas polyfunctional monocytes in adults, were only activated by bacterial not the fungal PAMP. Further, CB NK cells, like adults, express similar levels of Granzyme B [12, 56, 59] (Fig. 3C), making them a potent innate effector at birth. In contrast to our data, the one study [11] that probed the polyfunctionality of monocytes to LPS stimulation noted a lack of polyfunctional monocytes in CB; this difference with our data may relate to the combination of effectors measured. We probed polyfunctionality in terms of TNF-α, IL-6, IL-8, and IL-1β (Fig. 4), whereas the other report measured TNF-α, IL-6, and IL-12 [11].

Despite broad similarities in terms of the hierarchy of effectors secreted to bacterial and fungal PAMPs in particular (Fig. 1D), it was clear that CB and adult mononuclear cells differed in the magnitude of PAMP effector responses. Adults secreted significantly higher concentrations of TNF-α, IL-1β, IL-6, and CXCL-10 to bacterial, fungal and viral PAMPs (Fig. 1B and C), whereas CB secreted higher concentrations of IL-8, IFN-γ, IL-2, and IFN-α to the same PAMPs (Fig. 1B and C), broadly similar to previous reports [11, 34, 60]. The most striking difference we noted was the viral PAMP poly I:C. CB responded to poly I:C with a dominant IL-8 response whereas adults had a dominant CXCL-10 response (Fig. 1D). We confirmed this difference in the 6-h ICS assay and found that CXCL-10-secreting monocyte frequencies were significantly higher in adults (Fig. 5). However, the strikingly higher secretion of IL-8 by CB in the cytokine secretion assay (Fig. 1C) could not be explained by cell frequencies as CB and PBMC had similar IL-8 + cell subsets. Our data are consistent with the emerging importance of IL-8 in newborn immunity by orchestrating the recruitment and activation of neutrophils [5, 6, 61]. Indeed, more recent reports highlight that IL-8 expression in CB T cells may compensate for low level IFN-γ expression in the newborn [62–64]. Interestingly, SARS-CoV-2 lysate induced a similar combination of effectors in CB cells as poly I:C with IL-8 dominating indicating that CB cells are likely stimulated by SARS-CoV-2 lysate through a similar TLR-3 pathway as poly I:C. In contrast, adults differed in their response to poly I:C versus SARS-CoV-2 lysate; CXCL-10, the type 1 IFN pathway effector was predominantly secreted to poly I:C, whereas SARS-CoV-2 lysate response in adults was dominated by IL-6 (Fig. 1B and C). To the best of our knowledge, there are no reports highlighting the importance of IL-8 to viral PAMPs or indeed SARS-CoV-2 lysate activating CB cells in a similar manner to the canonical viral PAMP, poly I:C, though it is well established that SARS CoV-2 induces a potent pro-inflammatory response, which includes IL-6 in adults [32, 65]. These data also suggest that SARS-CoV-2 lysate likely does not serve as a TLR-3 agonist in adult cells. Instead, SARS-CoV-2 lysate is reported to trigger TLR 2 [66, 67]. PBMC responding to SARS-CoV-2 lysate with IL-6 as the dominant secreted effector is consistent with SARS-CoV-2 being profoundly pro-inflammatory in adults [65].

There could be several mechanisms that can potentially underpin CB versus adult differences in magnitude and combinations of effector responses to PAMP stimulation, as well as secreted concentrations and combination patterns of these cytokines differing between studies [60], including (i) PAMP dose, (ii) response time, and (iii) the nature of co-ordinately expressed cytokines, all of which can be impacted by exposure to other pathogens/ environmental cues. One marked difference was that adult PBMC secreted significantly higher concentrations of IL-1β (in particular) along with TNF-α and IL-6 to bacterial PAMPs (Fig. 1C). This difference could not be explained by differences in the cellular source of these effectors as high frequencies of monocytes expressing this combination of mediators were induced in CB cultures too (Fig. 3). This difference could also not be readily explained by probing the correlation matrix of secreted cytokines (Fig. 2), where IL-10 a key anti-inflammatory mediator and counter-regulator of TNF-α, IL-1β, and IL-6 was expressed in higher concentration by adults compared to CB cells; further, there was no evidence of negative correlation between TNF-α or IL-6 or IL-1β with IL-10 in CB cultures (Fig. 2). One possible explanation for this difference may relate to differential kinetics of TNF-α, IL-1β, and IL-6 expression following PAMP-stimulation by adults versus CB cells and/or the accumulation of other cross-regulatory mediators not included in our multiplex assay, and/ or the differential expression of these mediators by other innate cells (e.g. dendritic cells), not included in our immune staining panel. Similarly, the observation that CB can secrete low but detectable levels of IL-2 to PAMP stimulation, unlike PBMC, is potentially interesting and distinct from other studies showing IL-2 is not induced to a bacterial PAMP, e.g. LPS by either CB or adult PBMC [11, 34, 44] and could be a reflection of multiple factors, e.g. geography/exposure to environmental cues and/or lack of expression of a counterregulatory factor like IL-10 in CB, unlike PBMC (Fig. 1c).

By providing a comprehensive analysis of new-born innate immunity from India and placing this in context of adult PAMP responses from the same region, our study provides a strong foundation to explore mechanisms by which such immunity can be regulated/enhanced as well as provide a robust methodology to immune profile innate immunity during the life course, an area of significant global health importance given that infant mortality continues to be reported from the Indian subcontinent.

## Supplementary data

Supplementary data is available at *Clinical and Experimental Immunology* online.

uxae034_suppl_Supplementary_Materials

## Data Availability

The data presented in this study are available on request from the corresponding author.

## Abbreviations

AB: adult blood
CB: cord blood
CBMC: cord blood mononuclear cells
PAMPs: pathogen-associated molecular patterns
PBMC: peripheral blood mononuclear cells
TLRs: toll-like receptors.
