# Supplementary material for: Immune profiling reveals umbilical cord blood mononuclear cells from South India display an IL-8 dominant, CXCL-10 deficient polyfunctional monocyte response to pathogen-associated molecular patterns that is distinct from adult blood cells
Source: Clin Exp Immunol. 2024 May 2;217(3):263–78. doi: 10.1093/cei/uxae034 (PMC11310697; doi:10.1093/cei/uxae034)
Supplement: uxae034_suppl_Supplementary_Materials [file uxae034_suppl_supplementary_materials.zip › Tables/Supplementary Tables 1-2.docx]

**SUPPLEMENTARY FIGURE LEGENDS**

**Supplementary Figure 1: Subject recruitment and study protocol.** Consort flow diagram explaining clinical details of the study: on subject enrolment (n = number of subjects) in neonates and adults arms.

**Supplementary Figure 2**: **Spontaneous secretion of cytokines in cord blood (CB) and adult peripheral blood mononuclear cells (PBMC)**. Mononuclear cells isolated from infant cord (n=21) and adult blood (n=14) were kept in a CO2 incubator for 24 hours without stimulation after which supernatants were collected and cytokines were measured by a customized cytokine bead array to study the expression of 10 key innate and adaptive analytes. (A) The absolute concentrations of analytes in cord blood (CBMC in pink) and adult blood (PBMC in blue) for no stimulation control.

**Supplementary Figure 3**: **The representative sequential gating strategy for CBMC with LPS stimulation showing all the studied immune cell subsets.** Cells were stimulated with 1ng/ml LPS for 6 hours after which cells were stained with a panel of antibodies to study the expression of IL-1β, IL-8, IL-6, TNF-α, IL-17, IL-10 and IFN-γ. Unstimulated cells were used as negative controls. (A) Sequential gating strategy for arriving at CD3-CD16^-^CD56^-^HLA^-^DR^+^CD14^+^ cells and Sequential gating strategy for NK, NKT and gd T cells. (B) Overlay of unstimulated and LPS stimulated sample to show the expression of cytokines in HLA-DR^+^ cells.

**Supplementary Figure 4: Assessment of PAMP-specific immune cell subset by flow cytometry.** CBMC (n=21) and adult PBMCs (n=6) were stimulated with 1ng/ml LPS for 6 hours after which they were stained with a panel of antibodies to study the expression of IL-1β, IL-8, IL-6, TNF-α, IL-17, IL-10 and IFN-γ. Unstimulated cells were used as negative controls. (A) Representative expression of cytokines in CBMC and PBMC immune cell subsets studied in No-stimulation control. (B) Representative expression of cytokines in immune cell subsets studied after LPS stimulation. (C) Representative expression of Granzyme B in CBMC and PBMC of immune cell subsets studied in No-stimulation control.

**Supplementary Figure 5**: **Spontaneous cytokine-secreting cell frequencies differ between cord blood (CB) and adult peripheral blood mononuclear cells (PBMC)**. Mononuclear cells isolated from CBMC (n=21) and adult PBMCs (n=6) were stimulated with 0.25 x 10^6^ cfu/ml BCG or 1ng/ml LPS or 10^6^ cfu/ml *Candida albicans* for 6 hours after which cells were stained with a panel of antibodies to study the expression of IL-1β, IL-8 , IL-6, TNF-α, IL-17, IL-10, IFN-γ and IL-2. (A) Frequency of immune cell subsets producing at least one of IL-6, TNF-α, IL-1β, and IL-8 cytokines for No stimulation control in Cord blood (CBMC in pink) and adult blood (PBMC in blue) across immune cell subsets. (B) Frequency of immune cell subsets producing at least one of IFN-γ, IL-2, IL-17, and IL-10 cytokines for no stimulation control in cord blood (CBMC in pink) and adult blood (PBMC in blue) across immune cell subsets. Mann-Whitney test was used for comparisons shown in (A and B) P < 0.05 was considered significant. * P<=0.05, **P<0.01, ***P<=0.001, ****<=0.0001

**Supplementary Figure 6: Monocytes are the major cytokine-expressing immune cell subset across all PAMPs tested.** CBMC (n=21) and adult PBMCs (n=6) were stimulated with 0.25 x 10^6^ cfu/ml BCG or 1ng/ml LPS or 10^6^ cfu/ml for 6 hours after which cells were stained with a panel of antibodies to study the expression of IL-1β, IL-8, IL-6, TNF-α, IL-17, IL-10 and IFN-γ. No-stimulation cells were used as negative controls and subtracted from all the PAMP-stimulated samples. (A) Frequency of immune cell subsets producing innate cytokine IL-1β, IL-8, IL-6 and TNF-α after stimulating with BCG, LPS and *C. albicans* in cord blood (CBMC in pink) and adult blood (PBMC in blue). (B) Frequency of immune cell subsets producing adaptive cytokine IFN-γ, IL-17, IL-10, and IL-2 after stimulation with BCG, LPS and *C. albicans* in cord blood (CBMC in pink) and adult blood (PBMC in blue). Mann-Whitney test was used for comparisons shown in (A and B) P < 0.05 was considered significant. * P<=0.05, **P<0.01, ***P<=0.001, ****<=0.0001.

**Supplementary Figure 7**: **Representative sequential** **gating strategy for CBMC with poly I:C stimulation showing all the immune cell subsets studied.** Cells were stimulated with 100ug/ml poly I:C for 6 hours after which cells were stained with a panel of antibodies to study the expression of IL-1β, IL-8, IL-6, TNF-α, CXCL-10 and IFN-α. Unstimulated cells were used as negative controls. (A) Sequential gating strategy for arriving at immune cell subsets studied. (B) Representative staining for the effectors studied in unstimulated and poly I:C stimulated samples.

**Supplementary Figure 8: Paucity of CXCL-10 response in CB to viral PAMPs confirmed by cellular analysis.** CBMC (n=6) and adult PBMCs (n=6) were stimulated with 100ug/ml poly I:C or 1ug/ml SARS-CoV-2 lysate for 6 hours after which cells were stained with a panel of antibodies to study the expression of IL-1β, IL-8, IL-6, TNF-α, CXCL-10 and IFN-α. No-stimulation cells were used as negative controls and subtracted from all the PAMP-stimulated samples. (A) Frequency of immune cell subsets producing IL-1β, IL-6 and TNF- α after stimulating with poly I:C and SARS-CoV-2 lysate in cord blood (CBMC in pink) and adult blood (PBMC in blue). Mann-Whitney test was used for the comparisons shown. P < 0.05 was considered significant. * P<=0.05, **P<0.01, ***P<=0.001, ****<=0.0001

**Supplementary Table 1**: An 18-colour flow-cytometry staining panel to assess the cellular source of cytokines and chemokines induced by bacterial and fungal PAMPs.

**SUPPLEMENTARY TABLE 1**

| **Sl. No.** | **Marker** | **Flurochrome** | **Stain** | **Company** | **Cat.No.** | **Clone** |
| --- | --- | --- | --- | --- | --- | --- |
| 1 | Avid | V525 | CS | Invitrogen | L34957 | NA |
| 2 | CD56 | BUV737 | CS | BD | 612767 | NCAM16.2 |
| 3 | HLA-DR | PE-Cy5 | CS | BD | 555813 | G46-6 |
| 4 | gd TCR | PECF594 | CS | BD | 562511 | B1 |
| 5 | CD16 | APC-H7 | CS | BD | 560195 | 3G8 |
| 6 | CD14 | BV421 | CS | BD | 565283 | M5E2 |
| 7 | CD3 | BV-570 | IC | BL | 300436 | UCHT1 |
| 8 | CD4 | BUV397 | IC | BD | 563550 | SK3 |
| 9 | CD8 | BV-711 | IC | BD | 563677 | RPA-T8 |
| 10 | IFNg | APC | IC | BL | 506510 | B27 |
| 11 | TNFa | BV605 | IC | BL | 502936 | MAb11 |
| 12 | IL-8 | PerCPCy5.5 | IC | eB | 46-8088-42 | 8CH/BCH |
| 13 | IL-6 | PECy7 | IC | eB | 25-706942 | MQ213A5 |
| 14 | IL1-B | FITC | IC | BD | 340515 | AS10 |
| 15 | IL-10 | BV786 | IC | BD | 564049 | JES3-9D7 |
| 16 | IL-2 | PE | IC | BD | 559334 | MQ1-17H12 |
| 17 | IL-17 A | BV650 | IC | BD | 563746 | N49-653 |
| 17 | IL-17F | BV650 | IC | BD | 564264 | O33-782 |
| 18 | GzB | Alexa700 | IC | BD | 560213 | GB11 |

**Supplementary Table 2**: A 14-colour flow-cytometry staining panel to assess the cellular source of cytokines and chemokines induced by viral PAMPs.

| **Sl. No.** | **Marker** | **Flurochrome** | **Stain** | **Company** | **Cat.No.** | **Clone** |
| --- | --- | --- | --- | --- | --- | --- |
| 1 | Avid | V525 | CS | Invitrogen | L34957 | NA |
| 2 | CD56 | BUV737 | CS | BD | 612767 | NCAM16.2 |
| 3 | gd TCR | PECF594 | CS | BD | 562511 | B1 |
| 4 | CD16 | APC-H7 | CS | BD | 560195 | 3G8 |
| 5 | CD14 | BV421 | CS | BD | 565283 | M5E2 |
| 6 | CD3 | BV-570 | IC | BL | 300436 | UCHT1 |
| 7 | CD4 | BUV397 | IC | BD | 563550 | SK3 |
| 8 | CD8 | BV-711 | IC | BD | 563677 | RPA-T8 |
| 9 | TNFa | BV605 | IC | BL | 502936 | MAb11 |
| 10 | IL-8 | PerCPCy5.5 | IC | eB | 46-8088-42 | 8CH/BCH |
| 11 | IL-6 | PECy7 | IC | eB | 25-706942 | MQ213A5 |
| 12 | IL1-B | FITC | IC | BD | 340515 | AS10 |
| 13 | CXCL-10 | PE | IC | BD | 560088 | 6D4/D6/G2 |
| 14 | IFN-a | Alexa-647 | IC | BD | 555049 | 7N4-1 |

**SUPPLEMENTARY TABLE 2**
