# Supplementary figures and images for: Immune profiling reveals umbilical cord blood mononuclear cells from South India display an IL-8 dominant, CXCL-10 deficient polyfunctional monocyte response to pathogen-associated molecular patterns that is distinct from adult blood cells
Source: Clin Exp Immunol. 2024 May 2;217(3):263–78. doi: 10.1093/cei/uxae034 (PMC11310697; doi:10.1093/cei/uxae034)

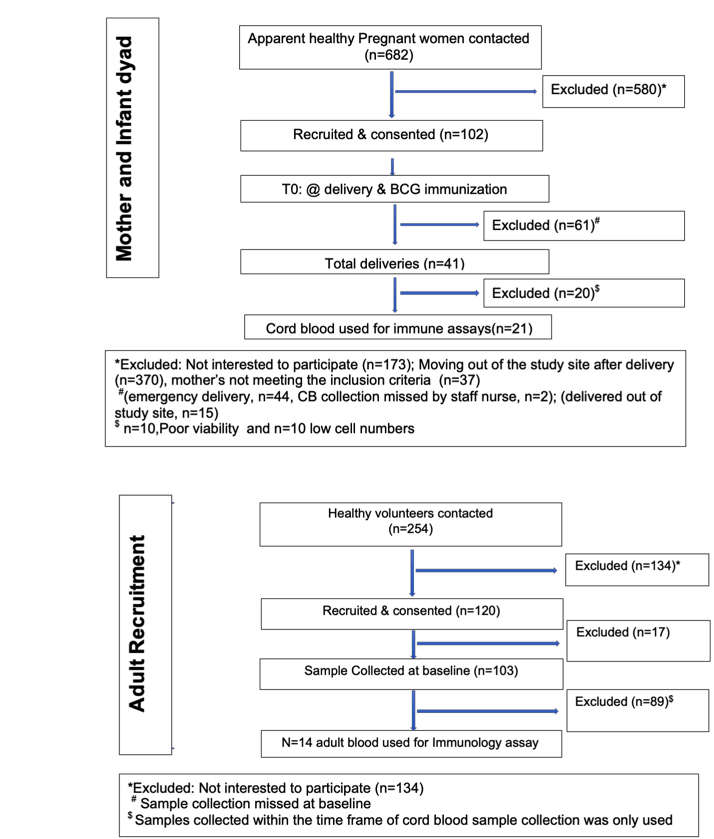

Supplement: uxae034_suppl_Supplementary_Materials [file uxae034_suppl_supplementary_materials.zip › Figures/Sup Figure 1.tiff]

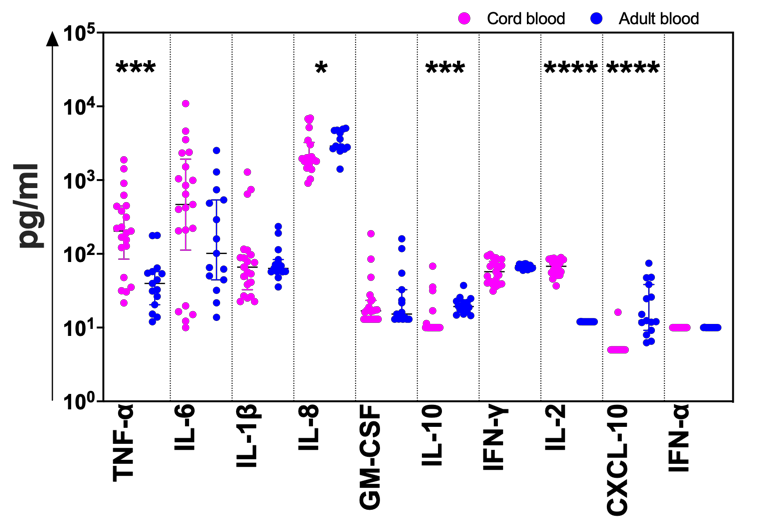

Supplement: uxae034_suppl_Supplementary_Materials [file uxae034_suppl_supplementary_materials.zip › Figures/Sup Figure 2.tiff]

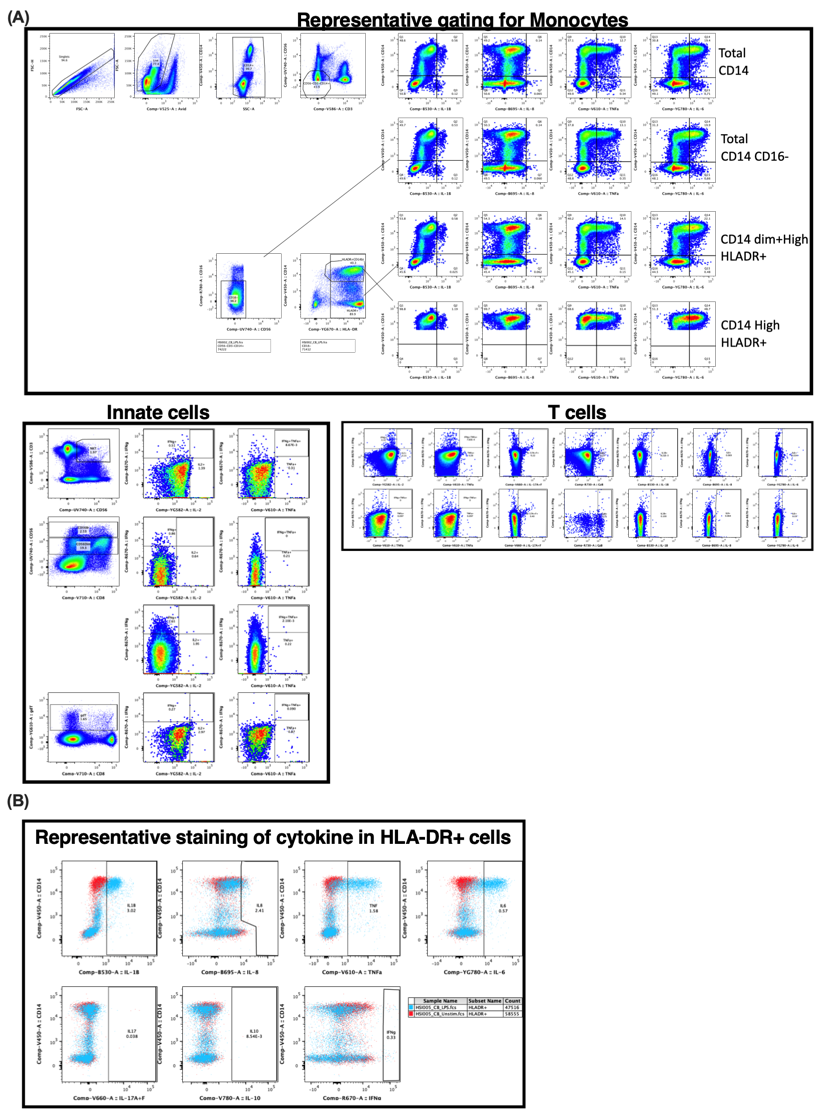

Supplement: uxae034_suppl_Supplementary_Materials [file uxae034_suppl_supplementary_materials.zip › Figures/Sup Figure 3.tiff]

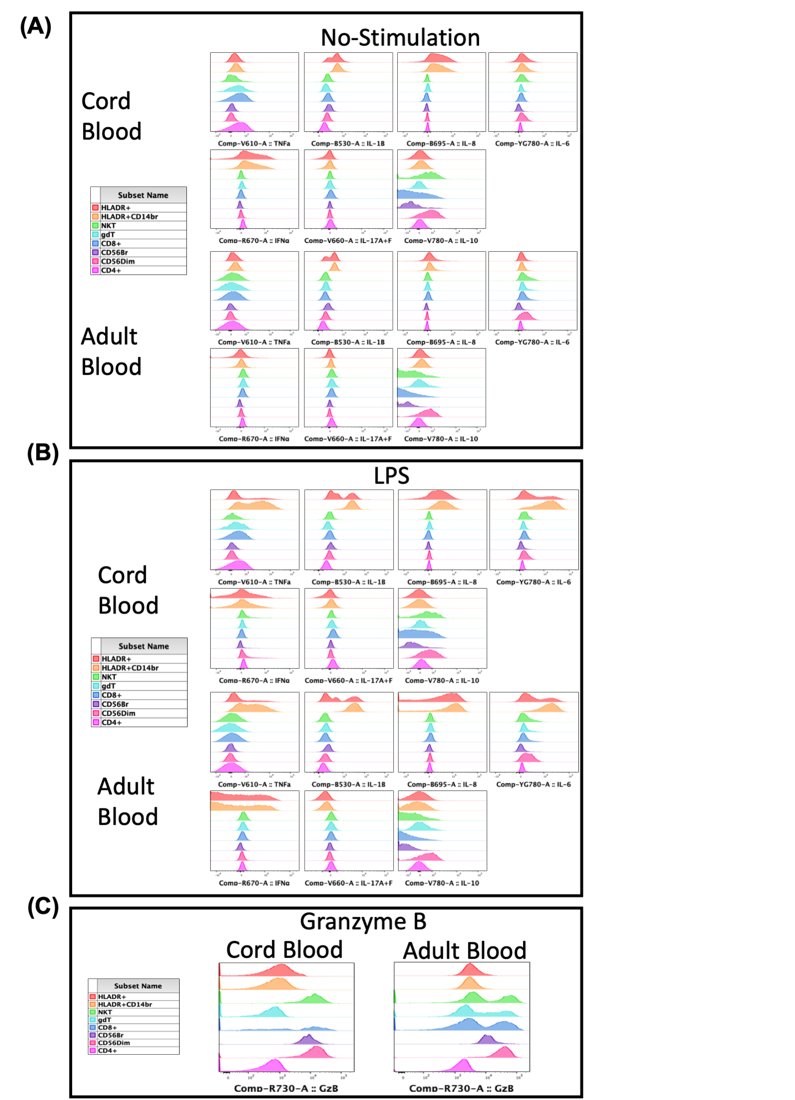

Supplement: uxae034_suppl_Supplementary_Materials [file uxae034_suppl_supplementary_materials.zip › Figures/Sup Figure 4.tiff]

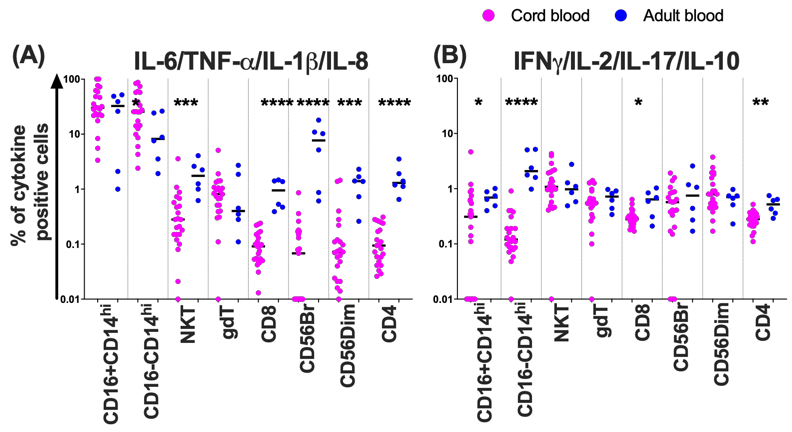

Supplement: uxae034_suppl_Supplementary_Materials [file uxae034_suppl_supplementary_materials.zip › Figures/Sup Figure 5.tiff]

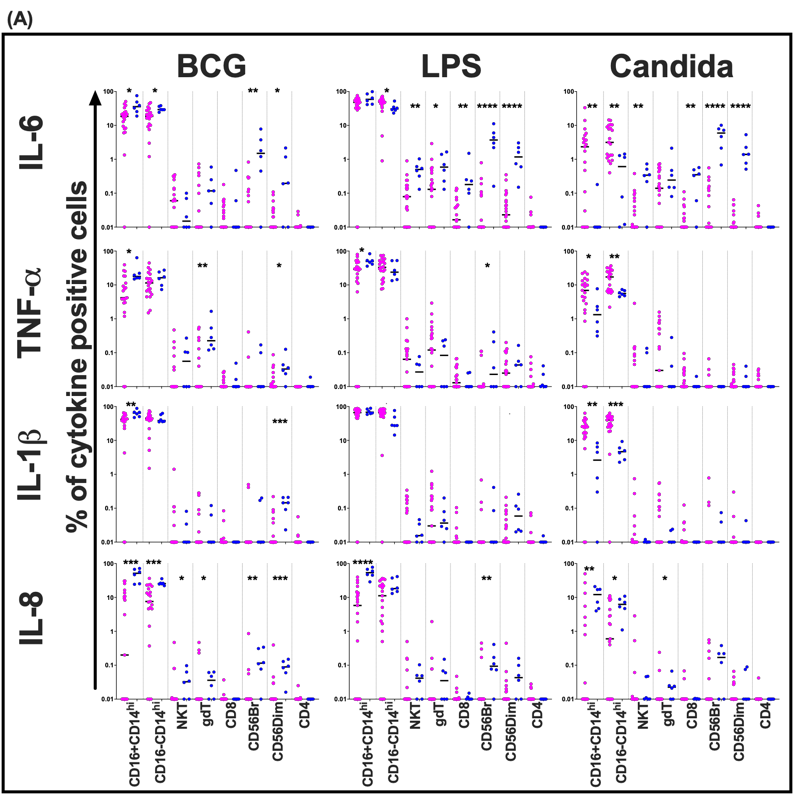

Supplement: uxae034_suppl_Supplementary_Materials [file uxae034_suppl_supplementary_materials.zip › Figures/Sup Figure 6A.tiff]

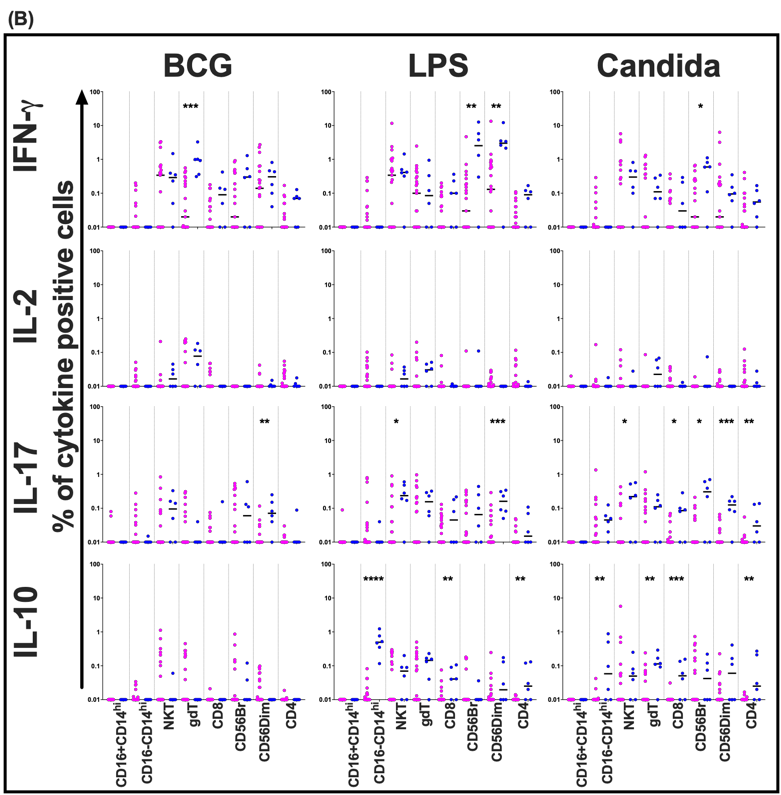

Supplement: uxae034_suppl_Supplementary_Materials [file uxae034_suppl_supplementary_materials.zip › Figures/Sup Figure 6B.tiff]

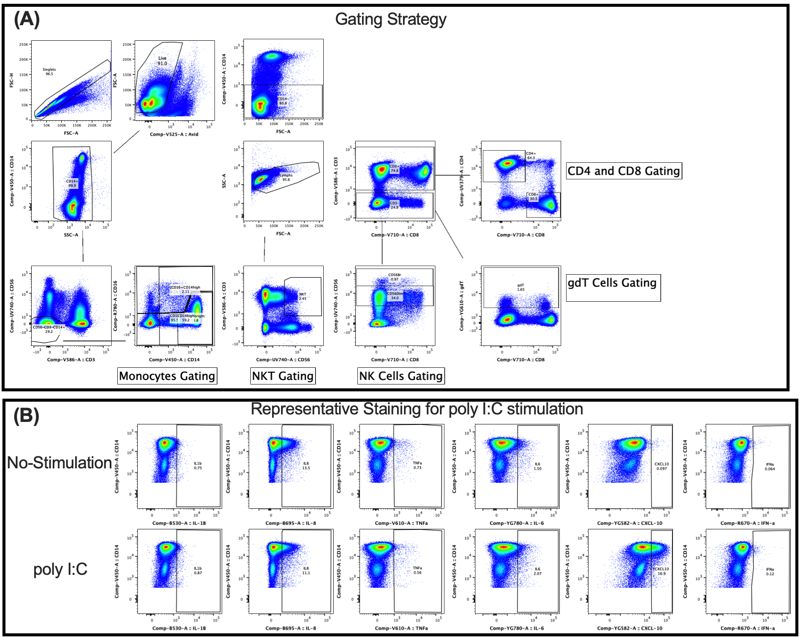

Supplement: uxae034_suppl_Supplementary_Materials [file uxae034_suppl_supplementary_materials.zip › Figures/Sup Figure 7.tiff]

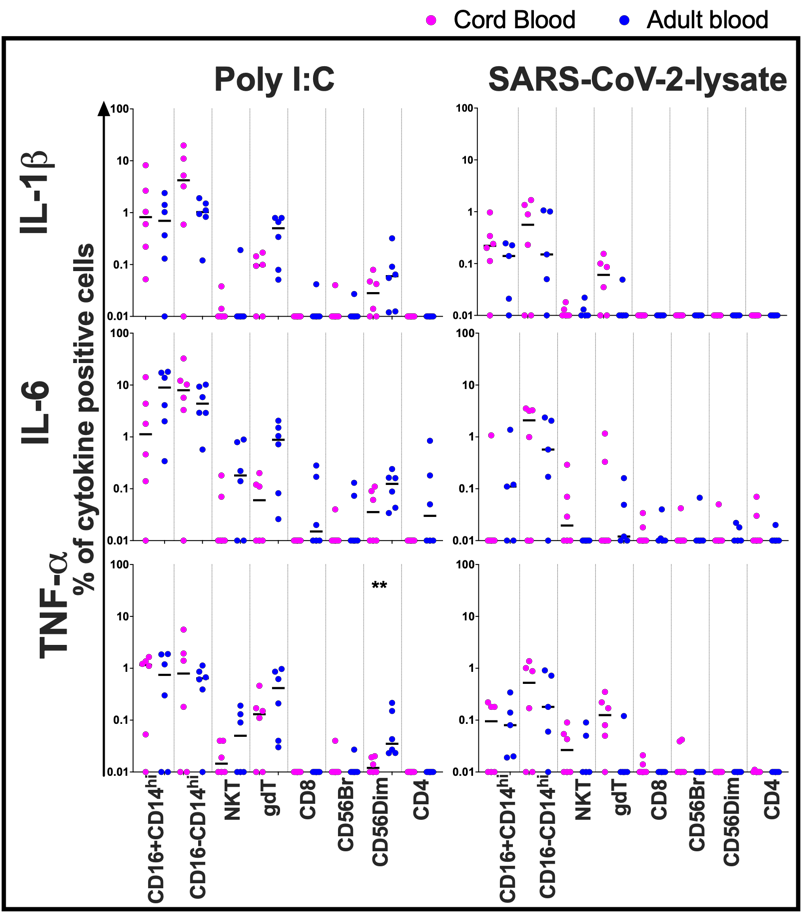

Supplement: uxae034_suppl_Supplementary_Materials [file uxae034_suppl_supplementary_materials.zip › Figures/Sup Figure 8.tiff]
